# Characterization of syntons across bacterial genomes using non-redundant genomic contexts

**DOI:** 10.1093/femsml/uqag033

**Published:** 2026-09-08

**Authors:** Alexandre Hervé, Jean Cury, Aude Bernheim

**Affiliations:** Institut Pasteur, Université Paris-Cité, CNRS UMR3525, Molecular Diversity of Microbes, 75015 Paris, France; Institut Pasteur, Université Paris-Cité, CNRS UMR3525, Molecular Diversity of Microbes, 75015 Paris, France; Institut Pasteur, Université Paris-Cité, CNRS UMR3525, Molecular Diversity of Microbes, 75015 Paris, France

**Keywords:** syntons, bacterial genomes, genomic context, gene clusters detection, genomic neighborhoods, phylogenetic redundancy, context residual entropy, antiphage defense systems

## Abstract

Bacterial genomes are dynamic, shaped by gene gain, loss, and horizontal transfer; yet selection preserves the colocalization of functionally coupled genes into recurrent modules known as syntons. Many large-scale approaches do not explicitly account for phylogenetic relatedness between genomes which can inflate support for associations that are simply shared by close relatives. To address this, we introduce a scalable framework that clusters gene-family occurrences by genomic neighborhood and counts co-occurrences across distinct genomic contexts, reducing redundancy from vertical inheritance and uneven database sampling without explicit phylogenetic reconstruction. Applied to 32 791 RefSeq genomes comprising 123 million genes, this approach identifies 15 million syntons across 779 000 synton families, characterizing their diversity and revealing structured associations among antiphage defense systems. Leveraging this context-aware framework, we also introduce context residual entropy to quantify gene families neighborhood variability. By prioritizing independent evolutionary events over redundant genome-level counts, our approach provides a scalable, phylogenetic tree-free view of modular organization across bacterial genomes.

## Teaser

By deduplicating gene associations based on their genomic context, we reveal the modular organization of bacterial genomes while distinguishing true functional coupling from simple inherited ancestry.

## Introduction

Bacterial genomes are highly dynamic, shaped by recurrent gene gain, loss, and horizontal transfer (Ochman et al. 2000, Koonin and Wolf 2008). This often results in open pangenomes in which only a limited set of core genes is conserved, while the accessory genome varies extensively, even between closely related strains (Tettelin et al. 2005, McInerney et al. 2017). Despite these variations, gene order is not random. Functional coupling, shared mobility, co-transcription, and positional constraints along the chromosome all contribute to maintaining genes in physical proximity (Dandekar et al. 1998, Rocha 2008, Marsh et al. 2013). To describe and quantify this non-random organization, multiple levels of genome organization have been proposed, including syntons, which are defined as sets of contiguous genes with conserved local organization across multiple bacterial genomes (Boyer et al. 2005). Syntons include classic co-transcribed operons such as *lac* or *trp* (Jacob and Monod 1961) and broader conserved gene neighborhoods such as ribosomal protein blocks (Sugita et al. 1997).

Detecting syntons systematically in bacterial genomes is challenging. Some methods focus on operon detection and typically use single genomes and intergenic distances, such as UniOP (Su et al. 2024) or transcriptional signals, like OpDetect (Karaji and Peña-Castillo 2025). For broader synton detection, conserved clusters are typically identified through multi-genome comparisons. CSBFinder (Svetlitsky et al. 2020) takes pre-computed gene-orthology-group annotations as input and identifies collinear blocks that recur, in the same order, across genomes. Spacedust (Zhang et al. 2025) instead identifies conserved gene clusters by combining structural homology with a statistical test on local gene order conservation, extending detection to homologs beyond the reach of sequence-based searches.

One challenge these methods must address is the lack of phylogenetic independence between genomes, which could lead standard co-occurrence models to overestimate the significance of conserved regions in closely related strains. To mitigate this, CSBFinder down-weights each genome’s contribution to the significance score by its overall gene-content similarity to the others, while Spacedust authors sub-sample genomes based on taxonomy, selecting one genome per genus. This approximates independence and keeps all-versus-all computations tractable. Both improve reliability, but neither fully resolves the following issue: when genomes are very close, large fractions of their gene order are shared by simple inheritance, and any approach treating each genome as an observational unit will struggle to separate this background conservation from genuine selective signal. Taxonomic subsampling sidesteps the problem by removing closely related genomes outright, but in doing so makes these methods blind to clade-specific innovations: a synton specific to *Escherichia* would necessarily be missed, since the necessary intra-clade comparisons are filtered out by design.

Here, we present a computational framework to infer syntons at scale without sacrificing clade-specific sensitivity, providing significance scores that explicitly correct for vertical inheritance and database sampling bias. This is handled by a deduplication heuristic that collapses observations of gene family colocalizations occurring in highly similar genomic environments into a single independent event, counting vertically inherited contexts once so that multiple observations reflect independent genomic events that gave rise to the unique contexts, such as rearrangements or horizontal transfers, and that the gene family colocalization persisted through.

## Results

### Syntons can be inferred from frequent gene families associations after sub-sampling families based on genomic context

We aimed to identify sets of contiguous genes with conserved local organization across multiple bacterial genomes, hereafter syntons (Boyer et al. 2005). In bacteria, purifying selection ensures that genes belonging to the same functional pair are rarely observed in isolation: if a recombination event splits a functional pair, the isolated genes often lead to a loss in fitness and are selected against. Consequently, we hypothesize that gene families co-occurring more frequently than expected by chance are functionally linked.

To measure those co-occurrences, we clustered Bacterial RefSeq genes into families at 50% identity (hereafter **fam50s**; Fig. 1A, panel 1) and counted how many times genes from each pair of families are found next to each other on genomes. However, direct co-occurrence counts between gene families across all genomes would heavily reflect the database’s sampling distribution. For instance, the many *E. coli* genomes in our dataset (2.7k, over 32 791 genomes in total) would artificially inflate the significance of any gene pair co-localizing in that specific species, regardless of whether the association is functionally driven or merely a result of shared vertical inheritance.

**Figure 1:**
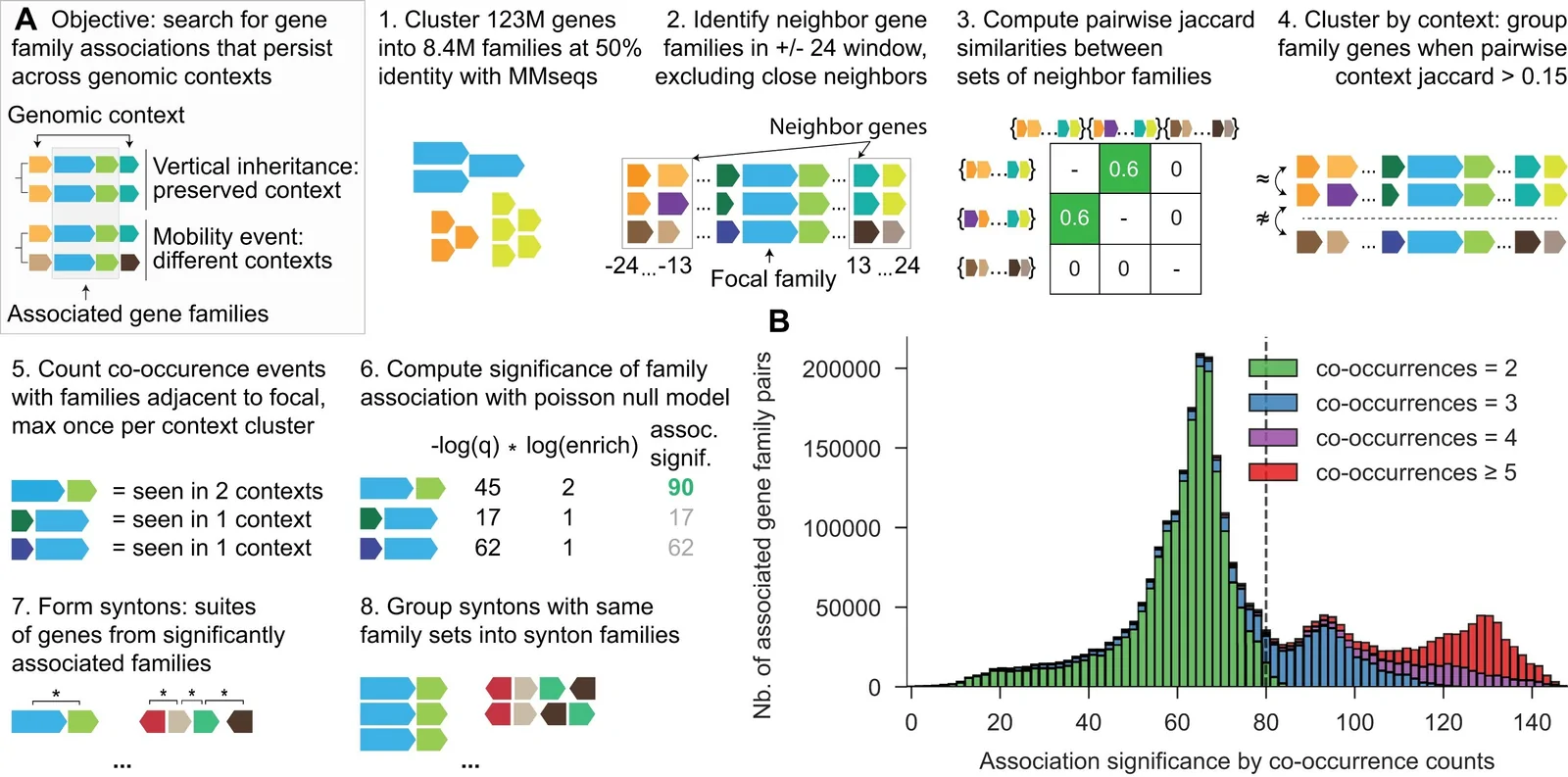
Context-based framework for identifying syntons from gene-family associations. (A) Overview of the pipeline used to detect syntons based on recurrent gene-family associations across diverse genomic contexts. The underlying principle is to identify gene-family associations that persist across multiple genomic neighborhoods. (1) All 123 M protein-coding genes are clustered into gene families at 50% sequence identity using MMseqs2. (2) For each occurrence of a focal gene family, neighboring gene families are identified within a ±24-gene window along the replicon, excluding the ±12 closest genes. (3) Genomic neighborhoods are represented as sets of neighboring gene families, and pairwise Jaccard similarities are computed between neighborhoods associated with the same family at 50% identity. (4) Neighborhoods are grouped into context clusters by single-linkage clustering, such that all neighborhoods within a cluster are connected by pairwise Jaccard similarities greater than 0.15. (5) For each pair of gene families, co-occurrence events are counted as the number of distinct context clusters of the focal family in which the partner family is observed, with each cluster contributing at most one event. (6) The statistical significance of gene-family associations is assessed using a Poisson null model, yielding a combined significance score that integrates association strength (fold enrichment as compared to Poisson lambda) and multiple-testing-corrected significance (q-value) (Methods). (7) Syntons are constructed within individual genomes as contiguous sets of genes whose corresponding gene families are pairwise significantly associated, allowing for a single intervening gene. (8) Syntons composed of the same set of gene families are grouped into synton families to identify recurrent architectures across genomes. (B) Distribution of association significance scores as a function of the number of supporting co-occurrence events, with colors indicating different event counts.

To alleviate sampling bias, we subsampled fam50s based on the neighboring genes, or genomic contexts, of their genes. The rationale is to count independent genomic events, like recombinations, that the association persisted through. The assumption is that each novel context in which we observe the genes of a family reflects one or more events that modified the genomic neighborhood and through which the association was retained. Over time, such newly formed contexts may be inherited vertically and sampled multiple times; by collapsing similar contexts into a single cluster, we aim to count each event at most once, independently from the spread of the clade that inherited the context.

Practically, we defined the genomic context of a gene as the set of gene families of its non-immediate neighbor genes, located from 12 to 24 coding sequences away on each side on the same replicon (Fig. 1A, panel 1). We then computed the pairwise Jaccard similarities between the genomic contexts to measure their similarities (panel 2), and linked in the same cluster any two genes whose pairwise Jaccard is above 0.15 (panel 3; Sup. Table 1). With this threshold, two genes sharing seven gene families or more among the 24 selected from their context cluster together (panel 4). The 12 closest neighbors were excluded from the neighbors sets to preserve our ability to reliably detect syntons up to size 12: without this exclusion gap, the conserved structure of an operon would act as a constant neighbor set, forcing all instances of a gene family to collapse into a single cluster, independently from the context around the operon. A comparison of the effect of gap and Jaccard threshold parameters is available in Sup. Fig. 1: while a smaller gap size and a lower Jaccard threshold, such as a gap of two genes and a Jaccard threshold of 0 would be too restrictive—preventing us from observing syntons of more than four genes, no strict biological measure dictates their values. Importantly, the changes in synton size distribution across parameter values are continuous and gradual, with no sharp transitions, indicating that the choice of parameters is not critical and that local variations around any given value would not qualitatively alter the results. We note, however, that as the Jaccard threshold approaches 1, genomic contexts become nearly unique, context-based deduplication loses its meaning, and sampling bias increasingly affects the results. Turning to the distribution of context clusters, the number of context clusters that we obtained per fam50 varies greatly, ranging from 1 to 124k (Sup. Fig. 2).

We then asked which gene-family associations were observed more frequently than expected by chance, once phylogenetic redundancy was removed. To do this, we first clustered the genes of each family based on their genomic context and sub-sampled one representative gene per cluster. We defined the A→B association count as the number of independent genomic environments, or context clusters, where a gene of family B was found adjacent to a sub-sampled gene of the focal family A (Fig. 1A, panel 5).

To assess the significance of these counts, we compared them to a Poisson null model. This model assumes that in the absence of selection, the arrival of a gene from family B into the neighborhood of family A occurs as a random, independent event with a constant probability across replicons. Because individual gene families are rare relative to the total number of available genomic slots, these events can be modelled as following a Poisson distribution with a rate λ representing the expected background co-occurrence rate based on the total frequency of family B on the replicons of A (Methods).

Using this null model, we calculated a p-value for each association, corrected for multiple testing with the Benjamini–Hochberg procedure (Benjamini and Hochberg 1995), and converted it into a significance score (Fig. 1A, panel 6; Methods; Sup. Table 2). We observed that a significance score above 80 typically corresponds to pairs found in at least three distinct genomic contexts (Fig. 1B). Consequently, we chose this threshold, alongside a maximum false discovery rate corrected p-value of 1e-5, to identify significant associations for the remainder of the study, yielding 1.03 M significant gene family pairs.

Overall, we obtained a significance score that allows us to rank and prioritize gene-family associations by their relative strength. It provides a practical means to identify the most robust functional links across bacterial genomes, though it is best interpreted as a comparative metric rather than an absolute threshold.

Finally, we used these significant pairwise associations to assemble larger structures: we defined a synton as a series of genes where each adjacent family pair is significantly associated (allowing a one-gene gap; panel 7; Sup. Table 3), and a synton family as a set of syntons sharing the same fam50s (Fig. 1A, panel 8; Sup. Table 4). Scanning 123 M genes from 32 791 genomes of the RefSeq prokaryotic database (Methods), we obtained 15 M syntons distributed into 779k synton families, with an average of 19.2 syntons per synton family.

### Syntons vary in size, genome occupancy, abundance, and taxonomic distribution

To examine the properties of the 15 M syntons identified across 32 791 bacterial genomes (Fig. 2), we began by asking how large these modules typically are. Quantifying the number of genes per synton revealed an exponentially shaped size distribution, with a median of three genes and a decay rate of 0.682 (R^2^=0.998) (Fig. 2A). This distribution should be interpreted in light of the context definition used here: because neighborhood similarity is computed while excluding only the ±12 closest genes, the method is inherently less sensitive to syntons extending beyond this scale. The observed size distribution therefore reflects the prevalence of compact gene assemblies within the detectable range rather than the full upper bound of synton sizes.

**Figure 2:**
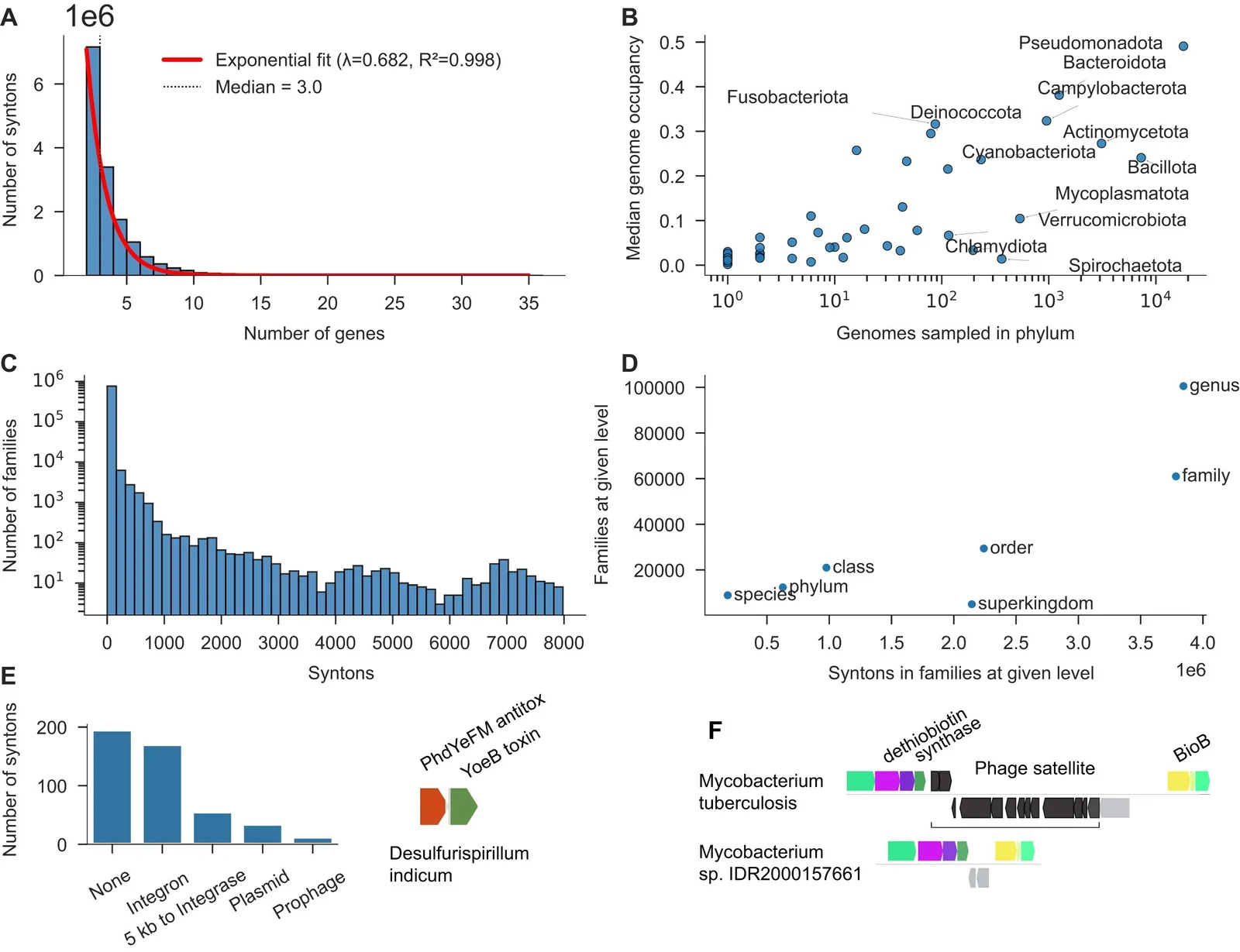
Distributions of syntons size, genome occupancy ratio, as well as synton family size and taxonomic breadth. (A) The number of genes per synton is exponentially distributed, with a median of 3 genes and a decay rate of 0.682 (R^2^=0.998) (B) Median proportion of the genomes occupied by synton in a phylum, per number of genomes sampled in that phylum (in log scale). The occupancy ratio by genomes count sharply rises at first, before slowing down. This reflects both the need for multiple genomes to significantly detect operons in a given phylum, and a trend towards a saturation effect at between 20% and 50% occupancy for above 10 and up to 10 000 genomes per phylum. (C) Distribution of the number of syntons per synton family, defined as sets of syntons composed of the same set of fam50s. The long tail of families containing up to 8000 syntons show the ability of the method to capture recurrent gene associations. (D) Taxonomic breadth of synton families. For each family, the lowest taxonomic rank containing all genomes from that family’s syntons is reported. The taxonomic breadth ranges from species to kingdom, reflecting the ability of the method to capture conservation at multiple scales. Most detected syntons are intra-genus, but we were still able to detect intra-species syntons. (E) A YoeB/PhdYeFM toxin-antitoxin synton is found 233 times in *Chrysiogenota, Thermodesulfobacteriota*, and *Pseudomonadota* phyla. About half occur on mobile genetic elements, including integrons, prophages, plasmids, or in the vicinity of integrases. (F) A 14-gene synton (black) from a phage satellite interrupts a pathogenicity-related biotin operon on a *Mycobacterium tuberculosis* genome (top). The synton is found 138 times on genomes of this species. A genome of *Mycobacterium* sp. IDR2000157661 without the satellite is shown for comparison (bottom), where similarly annotated genes are colored alike, except genes in grey that don’t match.

We then asked how much the method’s ability to detect syntons across bacterial phyla is driven by sampling depth. For each of the 48 phyla, we compared the number of genomes analyzed with the median fraction of genomes covered by syntons. We observed a rapid augmentation in genome coverage when the number of sampled genomes increased in a phylum, with no phylum with 10 genomes or less having a coverage above 15%, followed by a progressive slowdown as additional genomes were added; the phylum with the highest median genome occupancy is *Pseudomonadota*, reaching 48% (Fig. 2B). This pattern indicates diminishing returns in synton detection for well-sampled phyla, where additional sampling reveals new instances but yields progressively smaller gains in genome occupancy. Sampling density and genome occupancy are nonetheless not entirely correlated. For instance, *Fusobacteriota* and *Campylobacteriota* both display a coverage of around 30% whereas the former contains around a hundred genomes, and the latter around a thousand, ten times more. We conclude that our statistical power to detect associations increases with sampling density, an advantage of our approach over taxonomic subsampling, though clade-specific variations exist regardless of sampling density.

To assess how frequently identical synton architectures recur across genomes, we examined the distribution of the number of syntons per synton family, groups of syntons whose genes have the same set of fam50s. This distribution was highly heterogeneous, with a pronounced peak around 1 corresponding to singleton families and a long tail extending to families containing up to 8k syntons (Fig. 2C). This structure indicates that most syntons are rare, either by accident or because we detected truncated syntons by lack of statistical power, whereas a smaller number of synton architectures recur extensively across bacterial genomes.

Finally, we investigated the taxonomic depth at which synton families are shared, *i.e*. the smallest taxonomic level that contains all the syntons of a given synton family. Families spanned all taxonomic levels from species (8.9k families) up to kingdom (4.9k families), with the most families found at the genus level (100k families) (Fig. 2D). One example of a family shared at the kingdom level is the PhdYeFM/YoeB toxin-antitoxin system (Fig. 2E), which was detected 233 times overall: 231 times in *Pseudomonadota*, and once in each of the rarer phyla *Chrysiogenota* and *Thermodesulfobacteriota*. More recent, species-specific syntons are harder to detect: their identification requires several genomes of the same species to be compared against each other. Those genomes must also present enough diversity that syntons appear in distinct genomic contexts among them. Because genomic diversity is greater at the genus level, and because we have few species with multiple genomes—only 12% of species have three or more genomes in RefSeq, and 30% of genus have them—much more syntons are detected at the genus level compared to the species level. Still, when sampling is sufficient, the method is able to capture species-specific syntons of biological interest. We illustrate this in *Mycobacterium tuberculosis* (344 genomes in RefSeq) with a phage satellite synton shown to interrupt a pathogenicity-related biotin operon (Fig. 2F). The biotin operon is shown without the satellite in a genome of *Mycobacterium* sp. IDR2000157661 for comparison. These results demonstrate that our method can capture multi-gene assemblies at all evolutionary scales provided diverse enough genomes are available, including at the species scale.

### Variability of a gene family context is revealed by comparing its genes’ contexts with their sequence evolution

We hypothesized that gene families with higher genomic mobility should be observed across a broader diversity of genomic contexts than less mobile families, such as core genes. A naive measure of context diversity, the number of distinct context clusters (CCs) associated with a fam50, is however strongly confounded by phylogenetic breadth: families with wider taxonomic distributions can be expected to appear in more contexts regardless of their mobility.

To control for this effect, we subdivided each fam50 into sequence subfamilies at 95% identity (fam95s) and compared, for each fam50, the number of context clusters to the number of fam95s it contains. This effectively normalizes context diversity by sequence diversity among fam50s. When applied to three reference classes of gene families—transposases, defense-associated genes, and core genes—we observed marked differences in the ratio of context clusters per fam95 (Fig. 3A–C). Transposases exhibited the highest ratios (∼2.6 CC per fam95), consistent with extensive genomic reshuffling. Core genes showed much lower ratios (∼0.46 CC per fam95), nearly fourfold smaller, reflecting stable genomic embedding. Defense-associated families displayed intermediate values (∼0.83 CC per fam95), in line with their frequent localization in variable defense islands. These results indicate that the CC-to-fam95 ratio is informative about gene family mobility.

**Figure 3:**
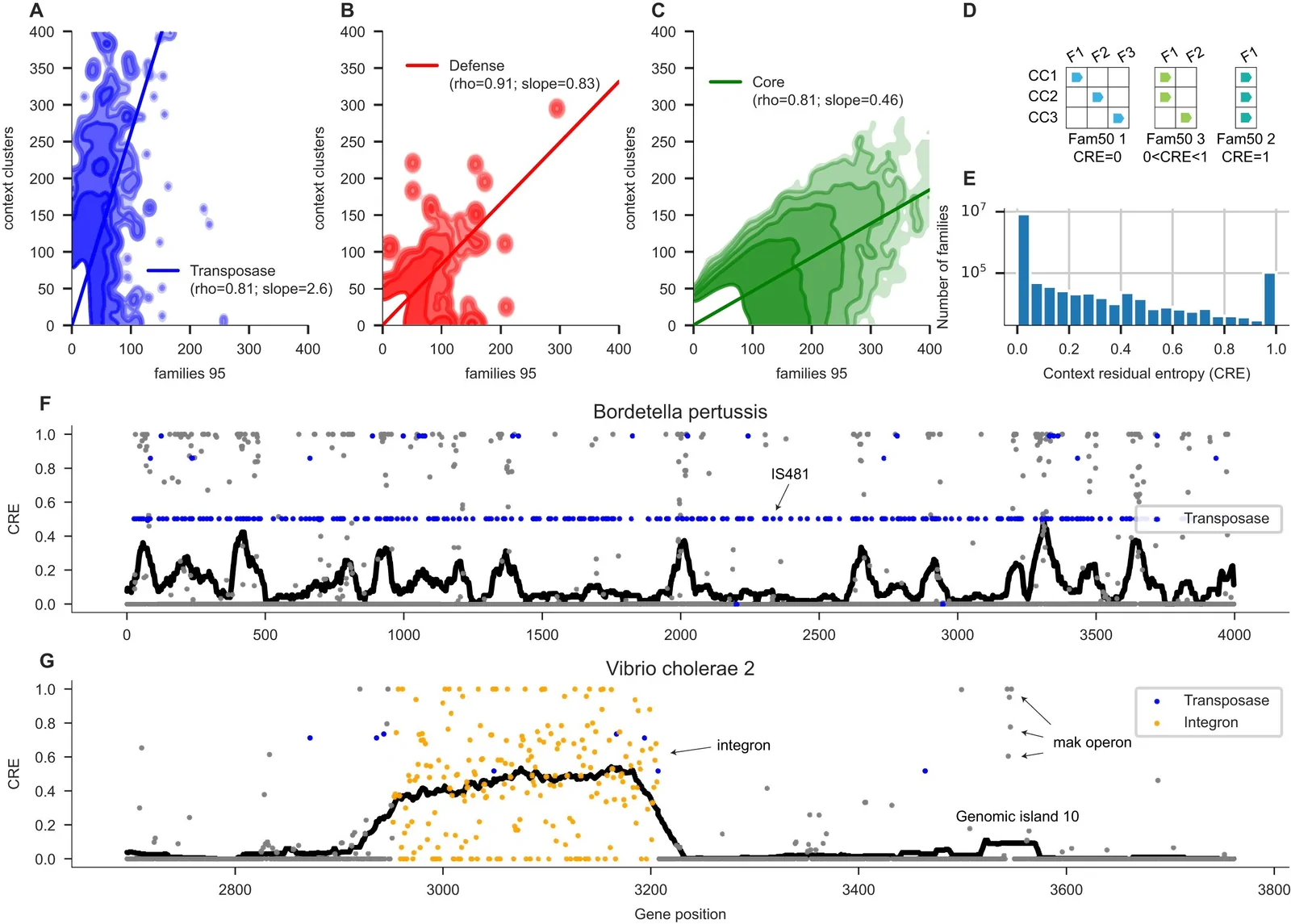
Context residual entropy (CRE) captures the relationship between sequence subdivision and genomic context diversity. (A–C) Joint density of the number of fam95 subfamilies and the number of context clusters per fam50 family. Lines indicate linear fits. Panels show representative subsets corresponding to families annotated as (A) Transposases, (B) defense-related genes, and (C) core genes. (D) Conceptual illustration of CRE for three example fam50 gene families. Rows correspond to neighborhood context clusters (CC) and columns to 95%-identity subfamilies (F) within each of the three fam50s represented. Filled cells indicate observed occurrences of genes from the fam50. CRE equals 0 when context is determined by the fam95 (left; example of a core family), equals 1 when contexts are independent of fam95 subdivision (right; example of a highly mobile family such as a transposase), and takes intermediate values when context diversity is partially explained by sequence subdivision (middle; example of a family with moderately diverse contexts). (E) Distribution of CRE values across all 8.8 M fam50 gene families from RefSeq. CRE values range from 0 to 1, reflecting varying degrees to which genomic-context diversity is explained by sequence subdivision. The peaks around 0 and 1 correspond to very stable (core-like) and very variable (transposase-like) contexts respectively. (F, G) Genomic distribution of fam50 genes along two chromosomes. Points indicate individual gene occurrences colored by annotation category (transposase or integron gene), with y value matching gene family CRE value. Black line: CRE rolling average with a smoothing window of 50. (F) *Bordetella pertussis* chromosome. The 221 occurrences of the IS481 transposase materialize as a horizontal line around 0.5. Several CRE peaks indicate other areas of genome variability. (G) *Vibrio cholerae* chromosome 2. The vibrio cholerae Integron (orange) is revealed by the high CRE values of its genes, indicating a high tendency to change their contexts, although rarer genes observed in a single context might have a CRE of 0. A secondary peak (right) corresponds to the enterotoxin-containing mak (motility-associated killing factor) operon, revealed by the diversity of contexts it explores.

However the CC-per-fam95 ratio does not capture how contexts are partitioned among fam95s. Specifically, it does not distinguish between families where each context is associated with a given fam95 and families with strong imbalance in the distribution. To address this, we introduced context residual entropy (CRE), defined as the Shannon entropy of the conditional distribution of genes in context clusters given their fam95 is known, normalized by the entropy of the distribution in context clusters itself (Methods). CRE values range from 0 to 1, enabling direct comparison across families; families observed in a single context were assigned a CRE of 0. CRE captures the fraction of context diversity explored by the genes of a family that cannot be explained by sequence subdivision of the family alone, *i.e*. by vertical inheritance in the absence of genomic rearrangements (Fig. 3D).

We computed CRE values for all fam50s in RefSeq and examined their genome-wide distribution (Fig. 3E; Sup. Table 5). The distribution of CRE values is strongly right-skewed, with most families clustered near 0 and a smaller but distinct peak near 1. Low CRE values correspond to families whose context diversity is fully explained by fam95 structure, which we interpret as genes embedded in conserved genomic regions, typical of core genome components. Conversely, higher CRE values identify families whose genomic contexts are less dependent on sequence variation, which is often the case for transposases for instance.

We next asked whether CRE could be used to localize areas of high variability along genomes. To this end, we mapped CRE values along the chromosomes of *Bordetella pertussis* and *Vibrio cholerae* (Fig. 3F–G). In *B. pertussis* (Fig. 3F), a rolling average of CRE values revealed multiple sharp peaks corresponding to clusters of high-CRE families, indicative of regions constituted of families with more variable contexts. Notably, a recurrent signal around CRE ∼0.5 corresponds to the 221 genomic copies of the IS481 transposase, a well-characterized transposase (McPheat et al. 1989) driving genome plasticity in this species. In *V. cholerae* (Fig. 3G), CRE profiling clearly delineates the canonical integron cassette (orange), as well as a secondary peak around position 3550 that corresponds to the mak (motility-associated killing factor) operon from genomic island 10, a polycistronic operon containing the virulence factor makA (Dongre et al. 2018, Herrera et al. 2022). Together, these analyses demonstrate that CRE provides a quantitative, normalized measure of gene family context variability and can help identify key variability-associated genomic features such as transposases, integrons, but also more subtle signals, such as the variability of the pathogenicity-associated mak operon context.

### Syntons contain many defense systems, isolated or in association

Defense systems comprise either single or multi-genes instances and are frequently found in variable genomic contexts, often near mobile genetic elements and defense islands. Because multi-gene defense systems are typically organized as operons, we expect them to be recovered in syntons; we therefore sought to assess how efficiently defense systems were recovered by our approach. To this aim we compiled the list of multi-gene system instances detected by DefenseFinder, a software that identifies antiphage defense systems on prokaryotic genomes (Tesson et al. 2022), and computed the proportion of these systems that were recovered fully within a single synton (Sup. Table 6). We then grouped instances by their DefenseFinder subtype among the 419 subtypes defined by DefenseFinder, each corresponding to a set of genes validated to confer a phage defense phenotype to their host, and, for each subtype, visualized the proportion of instances recovered in syntons as a function of the total number of instances detected in RefSeq (Fig. 4A). We observe that subtypes with less than 13 instances were not detected, whereas most subtypes with 13 or more instances were recovered, although with variable rates: SDIC1, SDIC4 and SDIC3 have similar high recall rates of around 90% despite having highly variable instance counts, from 12k to 56k. Restriction modification systems (RM) types I and II on the other hand are similarly abundant, with instance counts around the same order of magnitude from 18k to 26k, but have variable recall rates from 17% to 33%. Mok_Hok_Sok is the most abundant system that was not detected, with 2.8k instances. We also investigated the impact of the size of the defense systems on our ability to recover complete systems in a single synton (Sup. Fig. 3): systems larger than eight genes are generally harder to detect, though this likely reflects both a genuine limitation of the method and the lower abundance and greater gene-content variability inherent to larger systems. A notable exception is the Dpd system (12 or more genes), which is recovered with high frequency, suggesting that strong gene-order conservation can compensate for system size. We conclude that the method is well suited for the discovery of sufficiently abundant systems, with 78% of subtypes with more than 13 instances being detected in syntons at least once, but not well suited for exhaustivity, with a median recall rate per subtype with more than 13 instances of only 18% and low recall rates for syntons of more than eight genes. The limited statistical power can likely be imputed in great part due to the low granularity of the fam50s that served as a basis for the synteny analysis.

**Figure 4:**
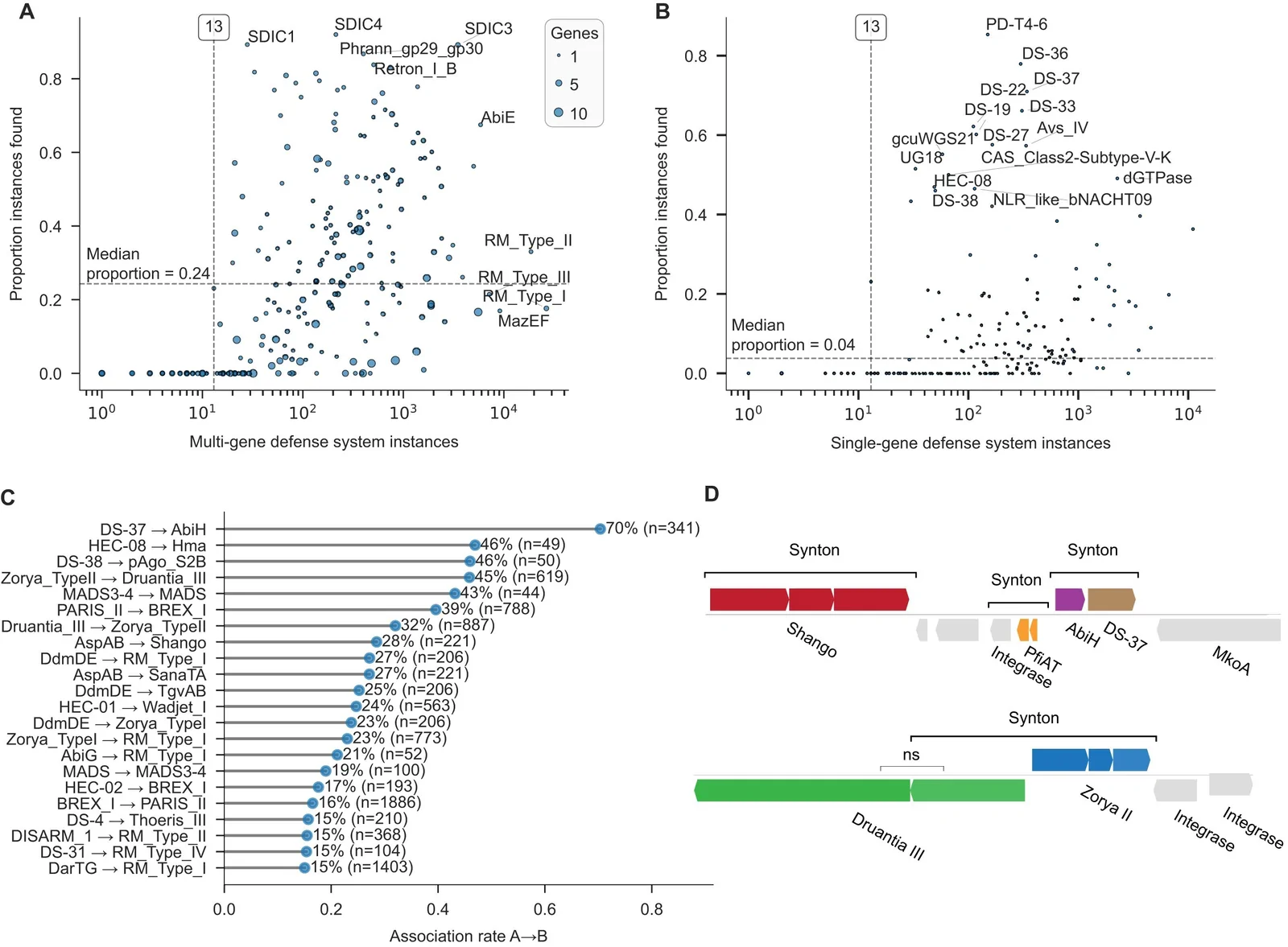
Occurrences and co-occurrences of defense systems within syntons. (A) Proportion of multi-gene defense systems in syntons. For each DefenseFinder subtype, the proportion of system instances fully recovered within a single synton is plotted as a function of the total number of instances observed in RefSeq. Each dot represents one subtype; selected systems are labeled. Dot diameter reflects the median number of genes in system subtypes’ instances. Systems with fewer than 13 instances are not detected, whereas most more frequent systems are recovered, albeit with variable recall rates. (B) Proportion of single-gene defense systems in syntons. For each DefenseFinder subtype annotated as single-gene, the proportion of instances found within syntons is shown as a function of total abundance in RefSeq. As in panel A, rare subtypes (<13 instances) are not found in syntons, while many more abundant single-gene systems are frequently found within syntons. (C) Frequent associations between defense systems in syntons. For each DefenseFinder subtype A, the association rate A→B indicates the fraction of total A instances that were found colocalizing with subtype B in syntons. Only the strongest associations are shown. Percentages indicate association rates, and “*n”* denotes the total number of instances of subtype A, in syntons or not. These associations highlight recurrent colocalization patterns between defense systems, consistent with stable functional pairings or higher-order defense assemblies. (D-E) Examples of genomic loci with defense-associated syntons. (D) A synton with AbiH and DS-37 finds itself near to other defense-related syntons: one containing PfiAT and an integrase, the other one containing the Shango defense system. (E) A synton contains Zorya II and the DruH gene of Druantia type III. The DruE helicase (left) was missed in the synton: the significance score of 74 for this DruE/DruH pair of fam50s is right below the 80 threshold chosen in this study despite co-occurrence observations in six independent contexts.

We then examined the proportion of single-gene defense systems that were found in syntons. To this end, we analyzed the fraction of recovered single-gene defense system instances as a function of the total number of instances observed in RefSeq, for each DefenseFinder subtype (Fig. 4B). Rare subtypes with fewer than 13 instances were again not detected, but 90 subtypes out of 187 were detected in syntons at least 10 times. Since those systems are single genes, their presence in syntons might come as a surprise. Several interpretations may account for this observation beyond statistical noise: single-gene systems might form systems with genes that are accessory to their own function. They might also be part of higher-order systems with complementary functions, including with other defense systems.

We next wondered if some defense systems would frequently colocalize with each other in syntons, revealing some possible interactions between defense systems that are not typically expected from systems in defense islands. To answer this question we computed, for each DefenseFinder subtype, the proportion of its occurrences associated with any other subtype, single gene or multi-gene, in syntons. We then searched for the most frequent associations, revealing some strong ties (Fig. 4C). Of the 341 instances of the single gene defense system DS-37 for instance, 240 (71%) were found to colocalize with AbiH (Forde and Fitzgerald 1999) in syntons, suggesting a potential synergy between DS-37 and AbiH. The association is weaker the other way around: those 240 colocalizations represent only 12% of the 1885 instances of AbiH. This imbalance could suggest that DS-37 might play an accessory role to AbiH. A DS-37–AbiH synton is illustrated in Fig. 4D alongside other multi-gene defense system syntons, PfiAT (Li et al. 2020) and Shango (Millman et al. 2022). The fact that each defense system sits in its own synton illustrates the ability of the method to isolate individual defense systems even in environments where they often collocalize with each other such as defense islands. A more balanced association is that of Zorya Type II with Druantia III (Doron et al. 2018), having 619 and 887 instances respectively, and seen together 284 times. This more balanced and partial association (46% in one direction, 32% the other way around) is indicative of a complementary role between the two systems rather than a scenario where one is accessory to the other (Fig. 4E). The DruE helicase of Druantia (left) is not part of the illustrated synton, the significance score of the association with its DruH neighbor being 74, slightly below the threshold of 80 required for significance. Such misses are inherent to threshold-based association detections, and illustrates the imperfect, statistical nature of the method. With 81 subtypes out of 519 being associated with another one in syntons more than 1% of the time, and with association frequencies up to 70% (Sup. Table 7), we conclude that a specific subset of defense systems tend to form syntenic associations with other systems, hinting at possible higher order interactions between defense systems.

## Discussion

We developed a framework to map syntons across bacterial genomes by identifying statistically significant gene associations while filtering out phylogenetic redundancy. By clustering gene occurrences with similar genomic neighborhoods, we avoid overcounting vertically inherited gene associations; instead, we approximate the number of independent evolutionary events that an association has undergone. Applying this strategy to 32 791 complete RefSeq genomes allowed us to characterize 15 million syntons, providing a high-resolution view of their size, genomic occupancy, and taxonomic distribution across all major bacterial phyla.

Beyond mere structural cataloging, we demonstrated that the relationship between a gene’s sequence diversity and its neighborhood variety captured by our CRE metric serves as a quantitative indicator of gene family context variabilities. Our analysis reveals that these syntons encompass a vast array of defense systems, often appearing as recurrent associations between distinct defense subtypes. Collectively, these findings reinforce the view of bacterial genomes as functional mosaics (Rocha 2008, Koonin 2009), dynamic arrangements reflecting a balance between long-term selective conservation and continuous genomic reshuffling.

Importantly, this framework is designed to scale to the full RefSeq database of 33k genomes and 123 M genes without requiring explicit phylogenetic reconstruction. While phylogeny-aware approaches can in principle distinguish vertical inheritance from independent association events, reconstructing and reconciling gene trees across tens of thousands of genomes and millions of gene families remains computationally prohibitive. Our context-based clustering therefore provides a pragmatic alternative that approximates evolutionary independence while remaining tractable for database-wide analyses.

Several limitations should be considered. First, statistical power depends on the definition of gene families. Families defined at 50% identity might be too narrow, leading to fragmentation of what would be a biologically coherent family into several 50% identity families. This can in turn reduce significance for some family associations, resulting in false negatives, as illustrated by the difficulty of the method to recover defense systems of eight genes or more in a single synton (Sup. Fig. 3). Second, sufficient sampling density within clades is required to detect recurrent associations at a given taxonomic level. Finally, although CRE captures differences in context–sequence coupling, it should not be interpreted as an absolute measure of mobility: CRE quantifies the extent to which genomic context diversification is decoupled from sequence diversification within a gene family. High CRE values therefore reflect context–sequence decoupling, which may arise from horizontal transfer but can also result from duplications, rearrangements, or other genomic processes.

Co-occurrence patterns between some defense systems also raise biological questions. Some systems experimentally validated as individually sufficient to confer phage resistance phenotype seem to behave like ancillary components within syntons. This suggests that the current experimental validation paradigm, based on single-system overexpression and plaque assays, might miss functional complementarities, and qualify ancillary genes as standalone defense systems. This concern is reinforced by recent work showing that overexpression of single defense systems substantially broadens their protection range against phages (Aframian et al. 2025), which could cause genes that contribute to defense only as part of a multi-gene assembly to appear sufficient when tested alone at non-native expression levels.

The recurrent co-localization of distinct defense subtypes further raises the possibility that certain systems form higher-order assemblies that travel together across genomes (Bernheim et al. 2020, Wu et al. 2024). Whether these associations only reflect functional complementarity at the level of individual genomes, or whether similar complementarities also exist at the population level, remains unclear. In other words, we might wonder if the complementarities observed within syntons may extend beyond the scale of single genomes and emerge as structured combinations of systems across strains within a species or ecological niche. Determining whether such population-level organization exists, and how it relates to genome-level modularity, will require integrating synteny analyses with population genomics and ecological data.

## Methods

### RefSeq dataset annotation

We downloaded all complete prokaryotic genomes from RefSeq (https://ftp.ncbi.nih.gov/genomes/refseq/bacteria/) in May 2023 (n = 32 791) together with their annotations and scanned them with DefenseFinder v2.0.2 to annotate known defense systems. The full proteome was clustered iteratively at various sequence identity thresholds (99%, 95%, 80%, and 50%) using MMseqs2 linclust. Throughout the manuscript, we used protein families clustered at an identity threshold 95% and 50% and are denoted as fam95 or fam50, respectively.

### Identification of genomic neighbors

For each occurrence of a gene family in a genome, we defined its genomic neighborhood as the set of fam50 of genes located within a window of ±24 coding sequence positions around the gene along the replicon, excluding the ±12 closest one on each side. This definition captures medium-range genomic context while minimizing trivial adjacency effects.

### Comparison of neighborhoods using Jaccard similarity

Each neighborhood was represented as an unordered set of gene families. Pairwise similarity between two neighborhoods belonging to the same gene family was quantified using the Jaccard index: $J( {A,\ B} )\ = \ | {A\ \cap \ B} |/| {A\ \cup\ B} |\ $where A and B are the sets of neighboring gene families associated with two independent occurrences of the same fam50. This measure reflects the similarity of their genomic environments.

### Context clustering of neighborhood instances

Neighborhoods associated with the same gene family were clustered based on their pairwise Jaccard similarity. Neighborhoods were grouped into context clusters such that all members of a cluster are connected through chains of pairwise similarities ≥ 0.15, corresponding to at least seven shared gene families within a 24-gene neighborhood. Each context cluster represents a distinct conserved genomic environment of the focal gene family.

### Counting independent co-occurrence events

For each pair of gene families (A, B), we counted the number of distinct context clusters of A in which genes of B were found immediately next to genes of A. This count represents the number of independent co-occurrence events supporting an association between the two families, reducing biases due to phylogenetic redundancy and uneven genome sampling. Context clusters, rather than individual gene occurrences, therefore define the unit of observation.

### Statistical assessment of gene-family associations

To test whether the observed number of co-occurrence events between two gene families exceeded random expectation, we modeled event counts using a Poisson null model. For each pair $( {A,\ B} )$, we computed the probability of observing at least $k\_obs$ events given an expected rate *λ *: $P(X\ \ge \ k\_obs\ |\ X\ \sim\ \mathit{Poisson}( \lambda ))$. The expected rate λ was derived from the overall frequencies of the two families, using context clusters as the unit of observation, and was computed as the sum of expected contributions across individual replicons. Formally for a given pair $( {A,\ B} )$:


\begin{eqnarray*}
{{\lambda }_{}}\ = \ 2 \times \mathop \sum \limits_{g\ \in\ \mathit{contexts}\left( A \right)}^{} n{{B}_r}\left( g \right)\ /\ {{L}_r}\left( g \right),
\end{eqnarray*}

where *contexts( A )\ *is the set of genes from A after deduplicating based on context clustering, $n{{B}_r}( g )\ $is the total count of genes of family B on the replicon where g is observed, ${{L}_r}( g )$ is the total number of genes on the same replicon, multiplied by two since there are two possibilities for B to neighbor A: after A or before A. Right-tailed p-values were adjusted for multiple testing using the Benjamini–Hochberg procedure (via the *statsmodels* Python package). Associations were further summarized using a fold-enrichment score and a combined significance score defined as:


\begin{eqnarray*}
\mathrm{Significance}\, \mathrm{score} &=& log2\left( {\mathrm{fold}\_\mathrm{enrichment}} \right)\\
&&\times \ \left( { - log10\left( {\mathrm{q}\_\mathrm{value}} \right)} \right),
\end{eqnarray*}

where $q\_value$ is the corrected $p\_value$. We retained only associations with a $q\_value$ inferior to 1e−5 and a significance score above 80.

### Construction of syntons

Syntons were defined on individual genomes as sequences of neighbor genes (with one gap allowed) whose corresponding gene families are pairwise significantly associated. Syntons therefore correspond to concrete genomic segments rather than abstract sets of gene families. The *networkx* Python package was used to assemble syntons as connected components in graphs of genes connected when their fam50s are significantly associated, and when they are at most two positions away on the same replicons.

### Synton families

Syntons composed of the same set of gene families at 50% sequence identity were grouped into synton families, enabling comparison of recurrent synton architectures across genomes.

### Context residual entropy

For each gene family at 50% identity, we quantified CRE as the ratio between the conditional entropy of genomic contexts given 95%-identity subfamilies and the marginal entropy of the distribution of genes into genomic contexts. For each gene of a given fam50, we defined a context label C corresponding to its neighborhood context cluster and recorded the associated 95%-identity subfamily *F95*. From the empirical joint distribution P$( {C,\ F95} )$ of context C and 95%-identity subfamily *F95* within a focal 50%-identity family *F50*, we computed the Shannon entropy $H( {F95} )$ and the joint entropy $H( {C,\ F95} )$, using natural logarithms. Conditional context entropy was defined as:


\begin{eqnarray*}
H(C\ |\ F95)\ = \ H\left( {C,\ F95} \right)\ - \ H\left( {F95} \right).
\end{eqnarray*}

Context residual entropy was then defined as:


\begin{eqnarray*}
CRE\ = \ H(C\ |\ F95)\ /\ H\left( C \right)\ if\ H\left( C \right)\ > \ 0\ \mathit{else}\ 0,
\end{eqnarray*}

$H(C\ |\ F95)$ varies between 0 and $H(C\ |\ F95)\ $when C is independent from *F95*, therefore *CRE* takes its values between 0 and 1. Values near 0 indicate that genomic contexts are largely predictable from 95%-identity subfamilies, whereas values near 1 indicate that contexts remain diverse even after conditioning on sequence subdivision.

### Computations

Except for the specifically mentioned python package, all computations were run using DuckDB. Analyzing the 32 791 genomes takes a few hours on 40 CPUs.

### Linear and exponential fits

Linear and exponential fits were computed using *scipy.stats.linregress* and *scipy.optimize.curve_fit*.

### MGE annotations

Transposases were annotated using Hmmer (Finn et al. 2011) and Pfam domains PF00872, PF01359, PF01498, PF01526, PF01527, PF01548, PF01609, PF01610, PF01710, PF01797, PF02281, PF02316, PF02371, PF02992, PF02994, PF03004, PF03017, PF03050, PF03108, PF03221, PF03400, PF03811, PF04195, PF04693, PF04754, PF04986, PF05699, PF05717, PF07592, PF08721, PF09039, PF10683, PF11427, PF12017, PF12596, PF12759, PF12760, PF12762, PF13007, PF13011, PF13022, PF13359, PF13542, PF13586, PF13610, PF13612, PF13613, PF13701, PF13737, PF13751, PF13808, PF13817, PF13842, PF13843, PF14198, PF14202, PF14319, PF14706, PF17489, PF17490, PF20167, PF20310, PF21517, PF21787, PF21788, PF21789, and PF21804. Prophages were annotated by geNomad v1.5.2 (Camargo et al. 2024), integrases with Pfam domain PF00589, integrons with IntegronFinder v2.0 with option –local-max (Neron et al. 2022), ICE elements with CONJscan (Cury et al. 2020), plasmids as annotated by RefSeq, phage satellites with SatelliteFinder (de Sousa et al. 2023).

### Core genes

Core genes were defined as genes from families (as clustered by MMseqs at 80% identity and 80% coverage) present in 99% of the genomes of any given species in RefSeq.

### Code availability

The full pipeline, documentation, and a small test dataset are publicly available at https://gitlab.pasteur.fr/mdm-lab/papers/bacterial-genome-syntons.

## Supplementary Material

uqag033_Supplemental_File

## Data Availability

Supplementary tables are available for download at https://doi.org/10.5281/zenodo.21099310.
