# Supplementary material for: Characterization of syntons across bacterial genomes using non-redundant genomic contexts
Source: Microlife. 2026 Sep 8;7:uqag033. doi: 10.1093/femsml/uqag033 (PMC13639185; doi:10.1093/femsml/uqag033)
Supplement: Supplementary file 1 [file uqag033_supplemental_file.docx]

# Supplementary Material

## Supplementary Tables

Supplementary tables are available for download at <https://doi.org/10.5281/zenodo.21099310>

**Supplementary table 1: context clusters**

The genes of each family at 50% identity that share similar genomic context are clustered together.

- Fam50: the family at 50% identity
- Context_cluster: the identifier of the context cluster per fam50
- Refseq: the RefSeq identifier of the gene

**Supplementary table 2: family associations**

This table describes the significance of associations between families at 50% identity.

- A50: base family
- B50: neighbor family
- K_obs: co-occurrence count
- Lambda: computed lambda rate for the Poisson model
- P_value: computed expected p_value from Poisson model
- Fdr_q_value: Benjamini-Hochberg corrected p_value
- Log2_fold_enrichment: log2(k_obs / lambda)
- Neg_log10_q: -log2(fdr_q_value)
- Sig_score: Log2_fold_enrichment * Neg_log10_q

**Supplementary table 3: syntons**

The list of genes in syntons

- Synton: the synton id
- Refseq: the RefSeq identifier of the gene

**Supplementary table 4: synton families**

Each synton is associated with the set of families at 50% identity of their genes, and synton with the same set are clustered together.

- Family: the identifier of the synton family
- Synton: the identifier of the synton

**Supplementary table 5: Context Residual Entropy**

- Fam50: the family at 50% identity
- Cre: its CRE value

**Supplementary table 6: defense systems in syntons**

- Subtype: defense system subtype
- Total_instances: total instances of this defense system in database
- Found_complete_instances: how many complete instances were found in syntons
- Recall: Found_complete_instances / Total_instances
- Mean_genes_per_instance
- Median_genes_per_instance

**Supplementary table 7: associations between DefenseFinder subtypes in syntons.**

For each DefenseFinder subtype, the proportion of its occurrences associated with other subtypes in syntons.

- Subtype_a: the subtype investigated
- Subtype_b: a subtype found next to subtype_a in syntons
- N_ab: the cooccurrences count
- N_a: how many instances of subtype a are found in syntons
- N_b: how many instances of subtype b are found in syntons
- assoc_rate_A_to_B: the proportion of a found next to b in syntons
- Recall_a: the proportion of total a instances found in syntons
- Total_a: the total number of a found in RefSeq
- Recall_b: the proportion of total b instances found in syntons
- Total_b: the total number of b found in RefSeq

## Supplementary Figures

**Supplementary figure 1**: Distributions of the number of genes in syntons families for various gap window and Jaccard threshold parameters. The total number of genes recovered in syntons for each parameter set is indicated as “n”. The gap size represents the number of genes that the Jaccard neighborhood windows are shifted off on each side of the focus gene. The distribution for parameters chosen in the study is highlighted with a black edge. We observe that the model is able to retrieve bigger syntons when either the gap or the Jaccard threshold increase, and that a gap 2 and a Jaccard threshold of 0 only allows recovering syntons of size 4 maximum. The total number of genes in synton increases with more permissive setups, from 9e6 with gap 2 and Jaccard threshold 0 to 9e7 with gap 12 and threshold 0.45. This ten-fold increase reveals that a large fraction of the additionally recovered genes likely represent spurious associations, as highly permissive settings reduce the stringency of context-based deduplication, allowing genomically ubiquitous genes to form artifactual syntons.

**Supplementary figure 2: Distribution of number of context clusters per fam50**

How many context clusters each family at 50% identity can be divided into.

**Supplementary figure 3: Percentage of multi-gene DefenseFinder systems entirely recovered in on single synton, per number of genes in the defense system**

The number of systems per gene count are indicated above the bars. DefenseFinder system subtypes with less than 20 occurrences were not taken into account. The high recall rates above a size of 10 correspond to high recall rates of the particularly well recovered Dpd defense system; the other 10+ gene systems, mainly CRISPR-Cas, are seldom retrieved as part of a single synton.
